# New Mutation in the Mouse *Xpd/Ercc2* Gene Leads to Recessive Cataracts

**DOI:** 10.1371/journal.pone.0125304

**Published:** 2015-05-07

**Authors:** Sarah Kunze, Claudia Dalke, Helmut Fuchs, Matthias Klaften, Ute Rössler, Sabine Hornhardt, Maria Gomolka, Oliver Puk, Sibylle Sabrautzki, Ulrike Kulka, Martin Hrabě de Angelis, Jochen Graw

**Affiliations:** 1 Institutes of Developmental Genetics, Helmholtz Zentrum München, Neuherberg, Germany; 2 Experimental Genetics, Helmholtz Zentrum München, Neuherberg, Germany; 3 German Mouse Clinic, Helmholtz Zentrum München, Neuherberg, Germany; 4 Federal Office for Radiation Protection, Department SG Radiation Protection and Health, Neuherberg, Germany; 5 Technische Universität München, Faculty of Life and Food Sciences Weihenstephan, Chair of Experimental Genetics, Freising-Weihenstephan, Germany; 6 German Center for Diabetes Research (DZD), Neuherberg, Germany; University of Delaware, UNITED STATES

## Abstract

Cataracts are the major eye disorder and have been associated mainly with mutations in lens-specific genes, but cataracts are also frequently associated with complex syndromes. In a large-scale high-throughput ENU mutagenesis screen we analyzed the offspring of paternally treated C3HeB/FeJ mice for obvious dysmorphologies. We identified a mutant suffering from rough coat and small eyes only in homozygotes; homozygous females turned out to be sterile. The mutation was mapped to chromosome 7 between the markers *116J6*.*1* and *D7Mit294*;4 other markers within this interval did not show any recombination among 160 F2-mutants. The critical interval (8.6 Mb) contains 3 candidate genes (*Apoe*, *Six5*, *Opa3*); none of them showed a mutation. Using exome sequencing, we identified a c.2209T>C mutation in the *Xpd*/*Ercc2* gene leading to a Ser737Pro exchange. During embryonic development, the mutant eyes did not show major changes. Postnatal histological analyses demonstrated small cortical vacuoles; later, cortical cataracts developed. Since XPD/ERCC2 is involved in DNA repair, we checked also for the presence of the repair-associated histone γH2AX in the lens. During the time, when primary lens fiber cell nuclei are degraded, γH2AX was strongly expressed in the cell nuclei; later, it demarcates clearly the border of the lens cortex to the organelle-free zone. Moreover, we analyzed also whether seemingly healthy heterozygotes might be less efficient in repair of DNA damage induced by ionizing radiation than wild types. Peripheral lymphocytes irradiated by 1Gy Cs^137^ showed 6 hrs after irradiation significantly more γH2AX foci in heterozygotes than in wild types. These findings demonstrate the importance of XPD/ERCC2 not only for lens fiber cell differentiation, but also for the sensitivity to ionizing radiation. Based upon these data, we hypothesize that variations in the human *XPD*/*ERCC2* gene might increase the susceptibility for several disorders besides Xeroderma pigmentosum in heterozygotes under particular environmental conditions.

## Introduction

ENU mutagenesis was frequently used to develop animal models for particular clinical phenotypes [1]. Since we are interested in genetic mouse models for eye diseases, we picked up a recessive ENU-induced mutant with small eyes, but also with rough coat (therefore it is referred to as *RCO015*).

Small-eye mutants in the mouse are genetically quite heterogeneous: dominant mutations include mainly *Pax6* (paired box 6), but also other genes like *Gjf1* (gap junction protein, ε1), *Cryba2* (βA2-crystallin), *Fgf9* (fibroblast growth factor 9), or *Pitx3* (paired-like homeodomain transcription factor 3, for a review, see [2]). Recently, we described two mouse mutants which have been identified by their smaller eyes in heterozygotes, but later in life or as homozygous mutants they developed cataracts; the affected genes are *Cryba2* [3] and *Lim2* (lens intrinsic membrane protein 2) [4]. From these genetic points of view, cataracts seem mainly a problem of terminal lens fiber cell differentiation; depending on the spatial and temporal expression pattern of the affected gene, it affects rather the primary or secondary lens fiber cells or both.

Lens fiber cell differentiation is a unique process, since it involves degradation of cell nuclei, mitochondria, including degradation of DNA—but the corresponding cells persists lifelong (the only other cell type loosing the organelles, are the erythrocytes—however, their lifetime is restricted in humans to ~115 days (varying between 70 and 140 days [5]). The process of nuclear breakdown in the lens starts in the mouse around birth for the primary fiber cells, and continues in the innermost secondary fiber cells (for a review, see [6] and references therein). As a result, we have an organelle-free zone in the center of the lens, and a cortical region with organelles at different stages of degradation.

Here, we describe for the first time a cataract (with a recessive mode of inheritance) caused by a mutation in the *Xpd/Ercc2* gene (Xeroderma pigmentosum complementation group D / excision repair cross-complementing rodent repair deficiency, complementation group 2). *XPD*/*ERCC2* belongs to the family of genes whose mutations lead in homozygotes to various forms of Xeroderma pigmentosum, here complementation group D (OMIM 278730). This well-known clinical trait is characterized by major UV-sensitivity of the skin; UV irradiation leads frequently to skin cancer. In humans, causative mutations are known affecting the genes *XPA-XPG and XPI* (for a review, see [7] and references therein); in mice several mutant lines are established in the homologous genes [8]. The high UV-sensitivity of the disease is caused by deficiencies in DNA-repair capacities and defines the major components of the nucleotide-excision repair system (NER; for a review, see [9]).

ERCC2 is well known as DNA helicase and involved in DNA repair [10]. Since the mutants show a nuclear and cortical cataract we hypothesize that the terminal differentiation process of the primary fiber cells (in the embryonic nucleus of the lens) and of the secondary fiber cells (at the lens cortex) is affected. Therefore, in the experiments reported here, we focus primarily on lens differentiation. Indeed, our experiments demonstrate that ERCC2 is expressed around birth in the central part of the lens; later in adolescence, we observe ERCC2 at the border of the differentiating lens secondary fiber cells to the organelle free zone.

Moreover, mutations in genes involved in DNA repair, frequently lead also to a higher sensitivity to DNA-damaging agents [11] like UV- or ionizing radiation. Even if van de Ven et al. [8] demonstrated a similar survival curve of mouse dermal fibroblasts (MDFs) from *Xpd* mutant mice and the corresponding wild-type cells, we tested the hypothesis of increasing sensitivity to ionizing radiation using the number of induced DNA double strand breaks indicated by the recruitment of γH2AX (phosphorylated form of the H2A histone family, member X) to the area of DNA damage [12] as parameter in murine lymphocytes from peripheral blood of heterozygous *RCO015* mutants. Our results indicate a higher sensitivity to ionizing radiation of lymphocytes from heterozygous mutants than the corresponding wild-type cells.

## Material and Methods

### Mice

Mice were kept under specific pathogen-free conditions at the Helmholtz Center Munich. The use of animals was in strict accordance with the German Law of Animal Protection and the tenets of the Declaration of Helsinki. The experiments were approved by the Government of Upper Bavaria (*Regierung von Oberbayern*; AZ 211-2531-55/01), and all efforts were made to minimize suffering. Euthanasia was performed by carbon dioxide inhalation. Male C3HeB/FeJ (C3H) mice were treated with ENU (90 mg/kg body weight applied by intraperitoneal injection in three weekly intervals) at the age of 10–12 weeks as previously described [13, 14] and mated to untreated female C3HeB/FeJ mice [15]. The offspring of the ENU-treated mice were screened at the age of 11 weeks for general dysmorphology features [16]. Scheimpflug imaging was performed according to Puk et al. [17] and slit-lamp analysis according to Kratochvilova [18].

### Linkage analysis

Homozygous mutant mice on C3H-background (G1) were outcrossed to wild-type C57BL/6J (B6) mice; C3HxB6 hybrid offspring (G2) were intercrossed, and the offspring (G3) derived from these matings were analyzed for their phenotype. DNA was prepared from tail tips of affected offspring of the third generation (G3). For linkage analysis, genotyping of a genome-wide mapping panel consisting of 153 single nucleotide polymorphisms (SNP) was performed using MassExtend, a MALDI-TOF (matrix-assisted laser/desorption ionization, time of flight analyzer) mass spectrometry high-throughput genotyping system supplied by Sequenom (San Diego, CA, USA [19]). Fine mapping was performed using 154 homozygous mutant mice and several microsatellite markers from the candidate region.

### Sequencing

Exome sequencing was performed by Otogenetics Corporation (Norcross, GA, USA) using DNA of one liver from a homozygous male mutant; bioinformatics analysis of the sequencing data were performed using the cloud analysis platform of DNAnexus (Mountain View, CA, USA). Exome sequencing was done in summer 2012; the mean coverage rate was 18.5x, and the amount of rRNA is given as 0.2%. After filtering the critical interval for homozygous mutations/polymorphisms predicted leading to an amino-acid exchange as the most likely causative event, we identified mutations/polymorphisms in 44 genes. As control, we had different mutants of the same genetic background, but with other mutations mapped to different chromosomes which excluded again 2 genes. Finally, we checked for the expression pattern of the candidate genes in the eye, leading eventually to *Ercc2* as the only candidate.

For re-sequencing and confirmation of the mutation in our breeding colony, genomic DNA was isolated from tail tips of JF1, B6, DBA/2J, CFW, and C3H wild-type mice or homozygous/heterozygous mutants according to standard procedures; cDNA was prepared from mRNA of isolated lenses. A 490-bp fragment was amplified from genomic DNA using the primer pairs Ercc2-L1 and R1 (Table 1) and digested by the restriction enzyme *Mwo*I. PCR was performed with a PTC-225 thermocycler (Biozym, Hessisch Oldendorf, Germany). Products were analyzed by electrophoresis on a 1.5% agarose gel. Sequencing was performed at GATC (Konstanz, Germany).

**Table 1:** Primers for PCR.

Name	Sequence (5’->3’)	Fragment size (bp)	Annealing temperature (°C)
Ercc2-L1	ACAAGCGTGGTAAGCTGCC	490 bp (gDNA)	64
Ercc2-R1	CTCTTGTAGATCGCTCCCTGC	320 bp (cDNA)	

### Analysis of lens development and maturation

Mouse embryos (or their heads) and eyes from embryonic or postnatal stages were analyzed histologically for eye pathologies. Tissues were fixed in Davidson solution and embedded in Technovit 8100 (Heraeus Kulzer, Wehrheim, Germany) according to the manufacturer’s protocol. Sectioning was performed with an ultramicrotome (OMU3; Reichert-Jung, Walldorf, Germany). Serial transverse 3-μm sections were cut with a glass knife and stained with methylene blue and basic fuchsin. The sections were evaluated with a light microscope (Axioplan, Carl Zeiss, Jena, Germany). Images were acquired by means of a scanning camera (AxioCam; Jenoptik, Jena, Germany) and imported into an image-processing program (Photoshop CS6, Adobe, Unterschleissheim, Germany).

### Immunohistochemistry

Eyes were embedded in paraffin and sections (8μm) were cut on a rotary microtome (Jung RM 2055, Leica, Nussloch, Germany). Antibodies against ERCC2 (Abcam plc, Cambridge, UK; order no ab102682; dilution 1:375) and γH2AX (Cell Signaling Technology, New England Biolabs GmbH, Frankfurt am Main, Germany; order no 9718, dilution 1:400) were used; binding to the corresponding antigen was visualized by a secondary antibody (Alexa Fluor488; Life Technologies GmbH, Darmstadt, Germany; dilution 1:250). Counterstaining of the cell nuclei was performed by DAPI (Sigma Aldrich Chemie GmbH, Taufkirchen, Germany; dilution 1:10.000). Stained sections were evaluated by a laser-scanning microscope (Olympus 1X81), equipped with imaging software Fluoview FV100 (Olympus Deutschland GmbH, Hamburg, Germany) and imported into an image-processing program (GIMP 2.8 or Photoshop CS6, Adobe Systems, Unterschleissheim, Germany).

### γH2AX assay in murine peripheral lymphocytes

Blood was taken from *vena cava anterior* subsequently after euthanasia of mice by carbon dioxide inhalation. Lymphocytes of 3 male wild type mice and 3 male RCO015 mutants were isolated from blood using MACS cell separation MicroBeads technique according to the distributers’ protocol (Milltenyi Biotech, Bergisch Gladbach, Germany).

Lymphocytes were irradiated with 1 Gy at the ^137^Cs-source HWM-2000 (Markdorf, Germany) at a dose rate of 0.47 Gy/min and subsequently incubated at 37°C for 6 h in pure Iscove’s Modified Dulbecco’s Medium (IMDM) containing 1% PenStrep (Biochrom, Berlin, Germany). 200,000 cells/100 μl PBS buffer were spun on top of a microscope slide (Hettich, Germany) prior to assay procedure. Cells were fixed in freshly prepared paraformaldehyde (2% in PBS) for 15 min and washed 3 x 5 min in Triton / PBS (0.15%), and subsequently blocked by 3x 10 min in PBS/BSA (1%). Slides were placed in a humid chamber and cells were overlaid with 75 μl of a 1:200 diluted γH2AX antibody (Cell Signaling Technology) and incubated over night at 4°C protected from light.

Following washing steps were performed at RT: 5 min PBS; 10 min PBS + Triton; 5 min PBS; 7 min PBS/BSA. 75 μl of a 1:1000 diluted sec. antibody (Alexa Fluor555-red/ Cell Signaling Technology) was again added onto the slide and incubated 45 min at RT. Cells were washed (2 x 5 min in PBS+Triton; 1x10 min in PBS; 1x7 min in PBS) and stained for 2 min at RT and again washed 2x 2 min in PBS. Cells were covered with 16 μl Vectashield/slide and sealed with a cover slip prior to foci analysis. Slides were analysed on a fluorescence microscope AxioImager.Z2 (Zeiss) using scanning and imaging function of the software Metafer 4 (MetaSystems, Altlussheim, Germany). Foci analysis of the acquired images was performed manually. For statistical interpretation the Welch’s two sample t-test was used.

### General

Chemicals and enzymes were from ThermoFisher (St-Leon-Rot, Germany), Merck (Darmstadt, Germany), or Sigma-Aldrich Chemie GmbH, Taufkirchen, Germany). Oligonucleotides were synthesized by Sigma Genosys (Steinheim, Germany).

## Results

### Detection of the *RCO015* mutants and identification of the underlying mutation

**Fig 1:**
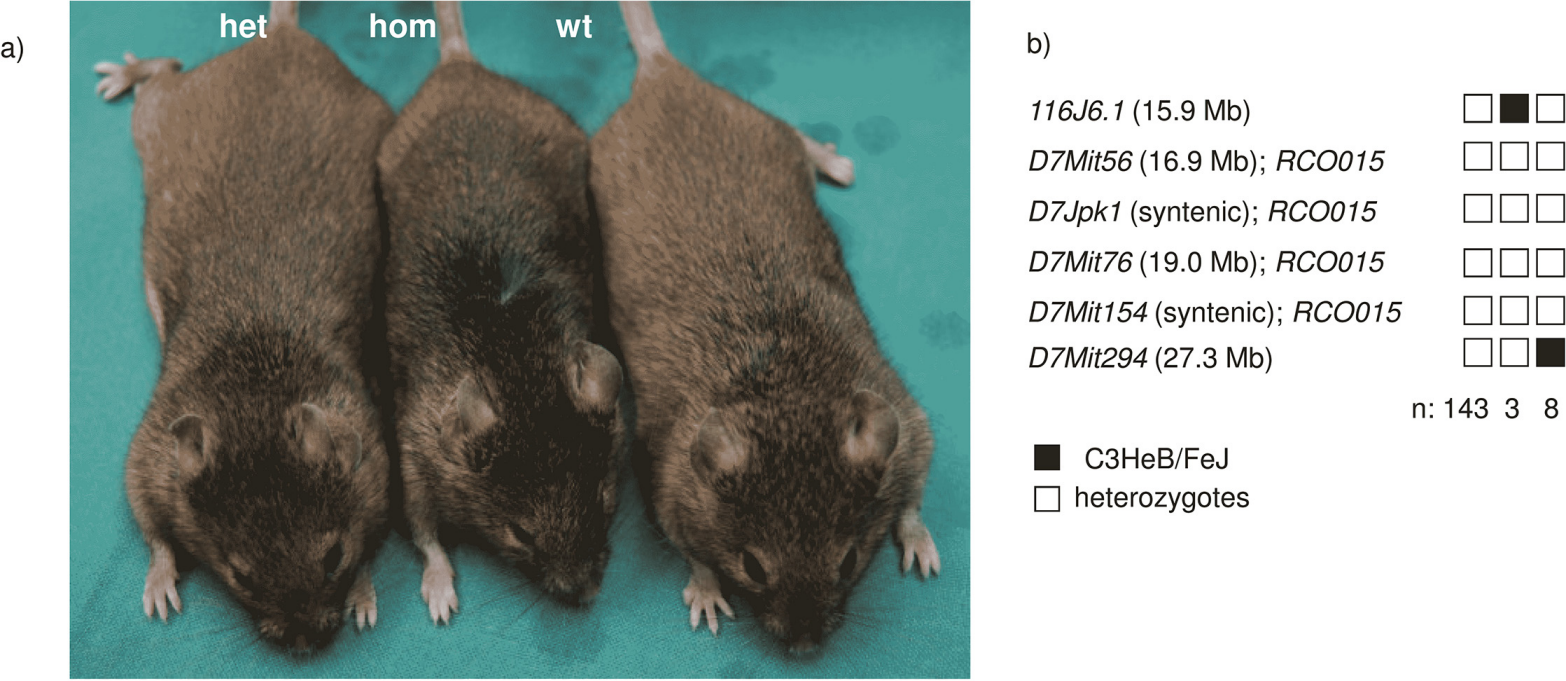
*RCO015* mutants and identification of the underlying mutation. a) A heterozygous (left) and homozygous (middle) *RCO015* mutant mouse at the age of 9 months compared to a wild type (right). The homozygous mutants are smaller and have rough hair and small eyes. b) Haplotype analysis defines the critical interval between the markers *116J6*.*1* and *D7Mit294* at mouse chromosome 7.

The *RCO015* mouse line was generated within our recessive ENU mutagenesis screen. According to the recessive trait, heterozygotes are without attracting attention, but homozygous mutant mice are characterized by rough coat (mutant symbol: *RCO015*). They were also remarkable smaller and had small eyes (Fig 1A). Homozygous female mutants never produced any offspring and were, therefore, considered to be sterile. The mutant line was kept by mating homozygous males with heterozygous females.

The *RCO015* mutation was mapped to mouse chromosome 7 close to the marker *rs4226424*. Subsequent fine mapping revealed a critical interval between the markers *116J6*.*1* and *D7Mit294*; markers within this interval (*D7Mit56*, *D7Jpk1*, *D7Mit76* and *D7Mit154*) did not show any recombination (Fig 1B). Because of this positional information of the mutation, functional candidate genes like *Apoe*, *Opa3* and *Six5* were analyzed, however, without differences to the reported wild-type sequences.

**Fig 2:**
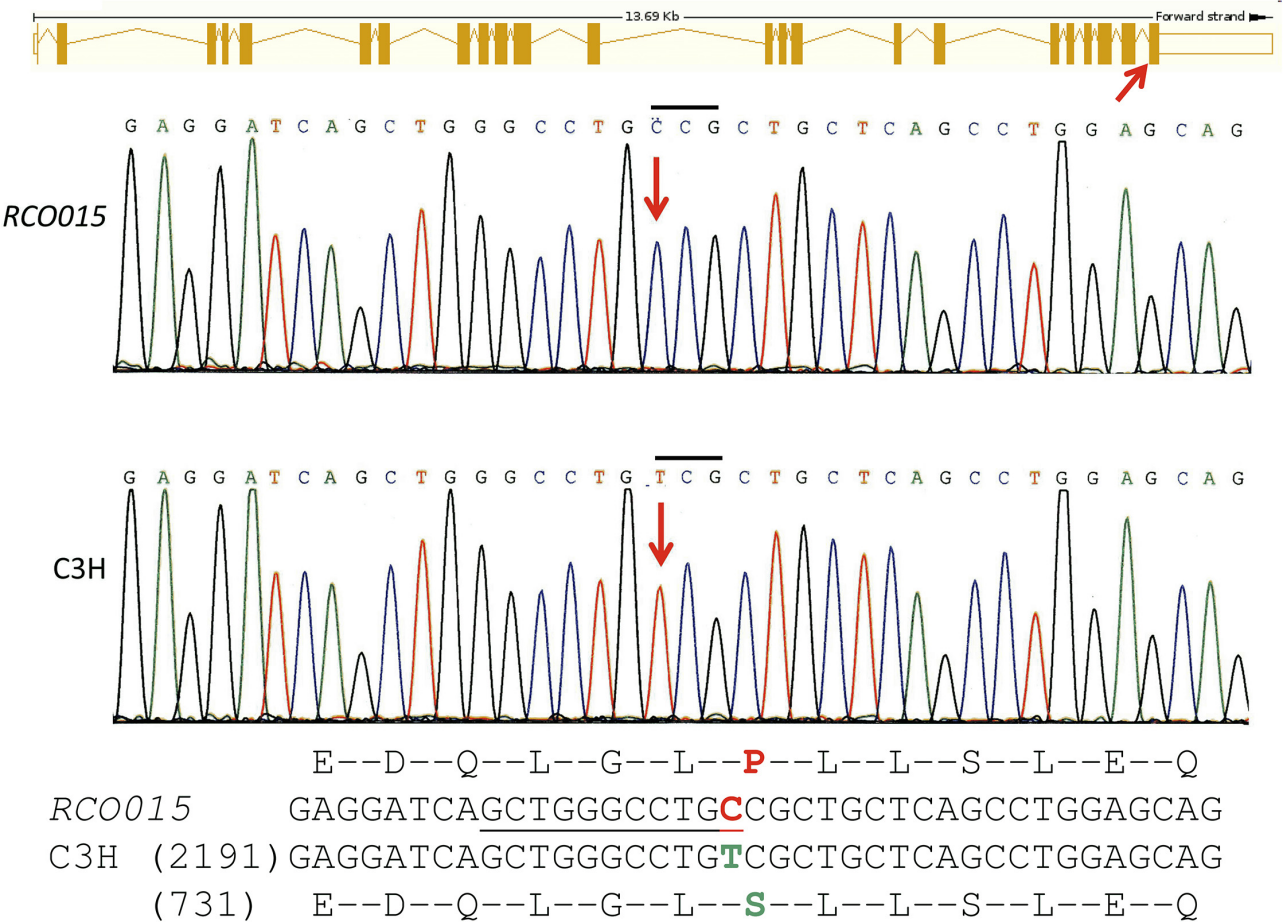
Sequence analysis of *Ercc2* gene. Schematic drawing of the mouse *Ercc2* gene (ENSEMBL); the red arrow points to the site of the mutation in exon 23. Sanger sequencing confirmed the exome sequencing data (c.2209T->C; red arrows); the frame of the sequence trace is indicated by placing a bar over the mutant codon. The changes in the amino acid sequence (Ser737Pro) are given below; the underlined DNA sequence demonstrates the new *Mwo*I restriction site in the mutants.

**Fig 3:**
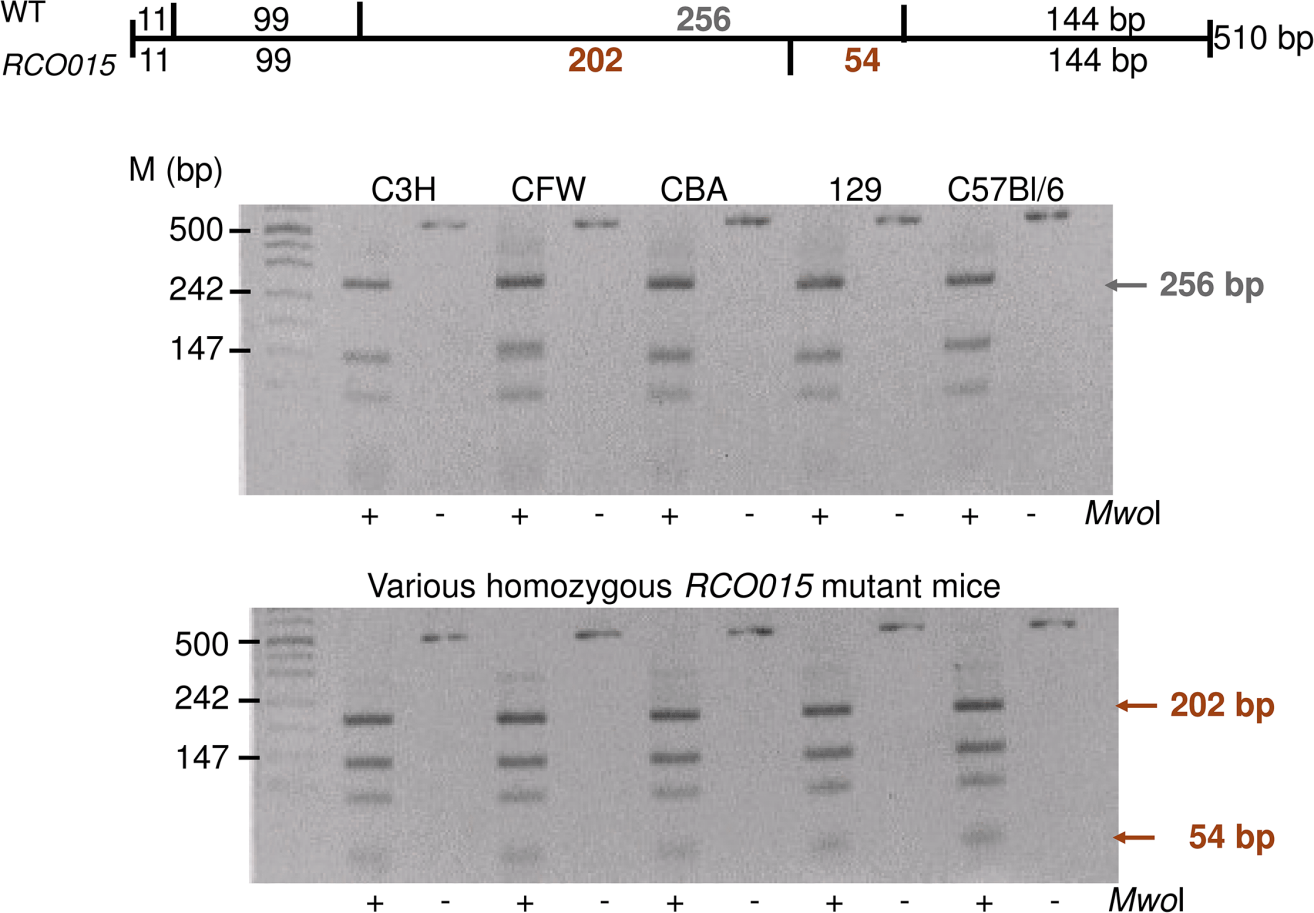
Confirmation of the *Ercc2* mutation by restriction digest. The novel *Mwo*I restriction site is present in all homozygous mutant mice tested. It is absent in 5 tested wild-type strains indicating that it is a mutation and no widespread polymorphism. The schema above the gels explains the digestion pattern of the fragment.

Exome sequencing detected the *RCO015* mutation in the *Xpd/Ercc2* gene (exon 23; Fig 2) at c.2209T>C resulting in a Ser->Pro exchange at amino-acid position 737 (Ser737Pro). The mutation was confirmed using classical Sanger sequencing (Fig 2) and by restriction digest using *Mwo*I, which is present in the mutants only (Fig 3). The PCR fragment remained intact in the 5 wild-type mice tested, but was digested in all 5 *RCO015* mice from our breeding colony. Therefore, we conclude that this missense mutation is a true mutation and no polymorphism.

GOR4, a secondary protein structure prediction program (https://npsa-prabi.ibcp.fr/cgi-bin/npsa_automat.pl?page=npsa_gor4.html), suggests a break within long-helical structure (30 amino acids) by 3 amino acids forming a random coil at the very C-terminal part of the protein. Concurrently, ProDom (http://prodom.prabi.fr/prodom/current/html/form.php) predicts a lower score for the helicase domain ranging from position 703 to 759. Moreover, S737 is a potential phosphorylation site like S740. We checked the probability of both to be phosphorylated and received a score of 0.195 for S737 and of 0.478 for S740 in the wild type; however, if Pro is present at pos. 737 as in the mutant, the score for S740 decreases to 0.162 indicating an interesting position effect. However, since all scores are below 0.5, phosphorylation at these sites does not seem to be very likely (http://www.cbs.dtu.dk/services/NetPhos/).

### Lens morphology and ERCC2 expression in the lens

**Fig 4:**
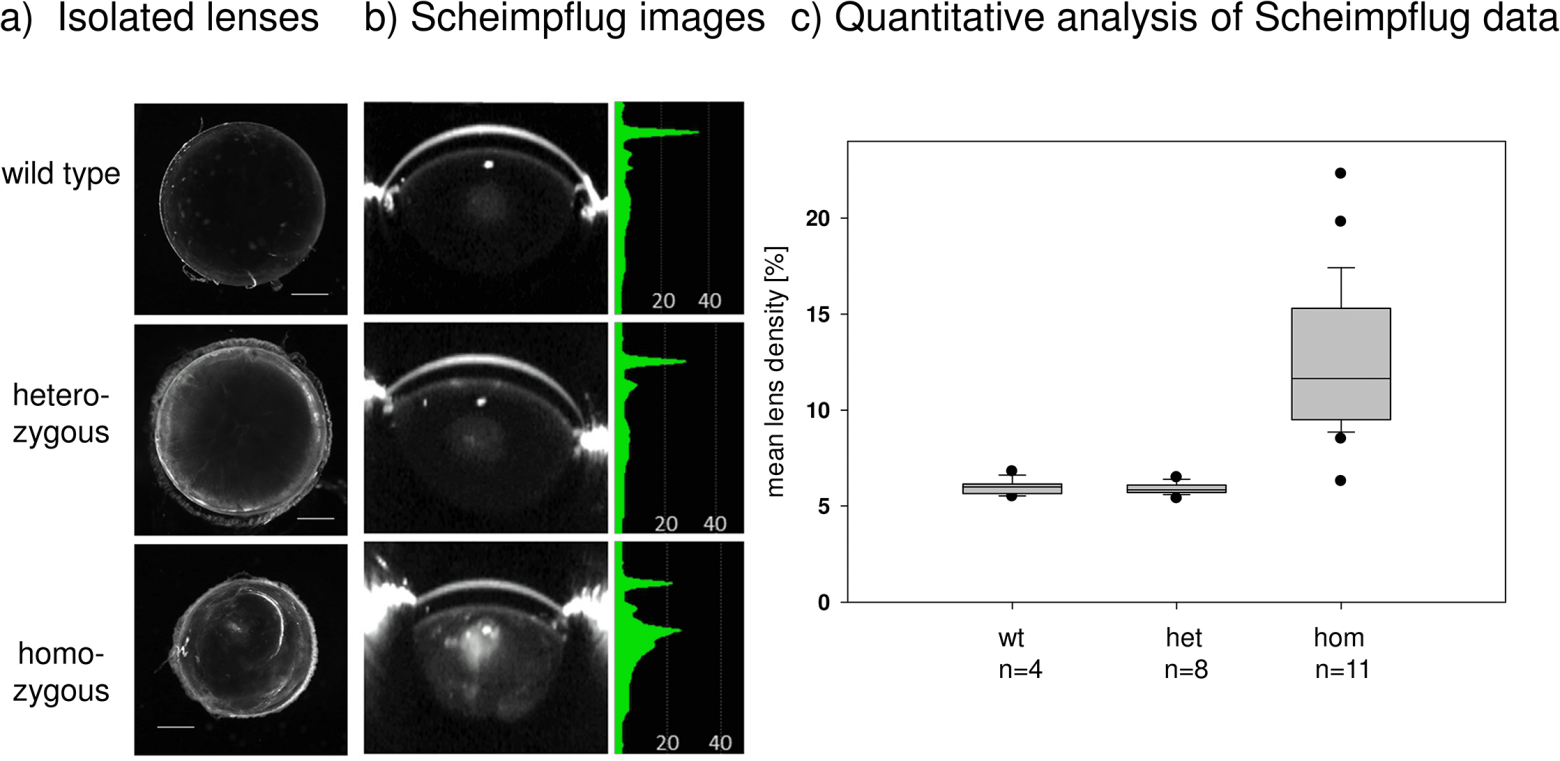
Cataracts in *RCO015* mutant mice at the age of 5 weeks. a) Lenses of wild types, hetero- and homozygous *RCO015* mutants are prepared and photographed. The lenses of wild types are completely clear; the lenses of heterozygous mutants demonstrate opacities at the capsule, and in the homozygous mutants clear boundaries in the cortical areas are observed in addition to the nuclear opacity. The lenses of homozygous mutants are smaller (bar: 500 μm). b) Scheimpflug imaging of the same lenses as shown in a) demonstrates the clear lenses in wild types and heterozygotes; the opacity in the nuclear region of homozygous mutants is clearly visible above the slightly opaque background of the entire lens. c) The quantitative data of the lens density of the Scheimpflug images are given in a box-and-whisker plot; the whiskers give the 1^st^ and 3^rd^ quartiles, and the bar in the middle of the box indicates the median of the lens density.

The *RCO015* mutants became interesting because of their small-eye phenotype as homozygotes. Isolated lenses demonstrated that they were cataractous in homozygous mutants and also smaller (Fig 4A and 4B). The cataract at the age of 5 weeks is characterized by an anterior-nuclear opacity and also by ring-like structures, which can be observed only in the isolated lenses but not in the Scheimpflug images. Quantification of the Scheimpflug data (Fig 4C) demonstrated an increase of the mean lens density from 6.0% in wild types and 5.9% in heterozygotes to 12.7% in the homozygous mutants.

**Fig 5:**
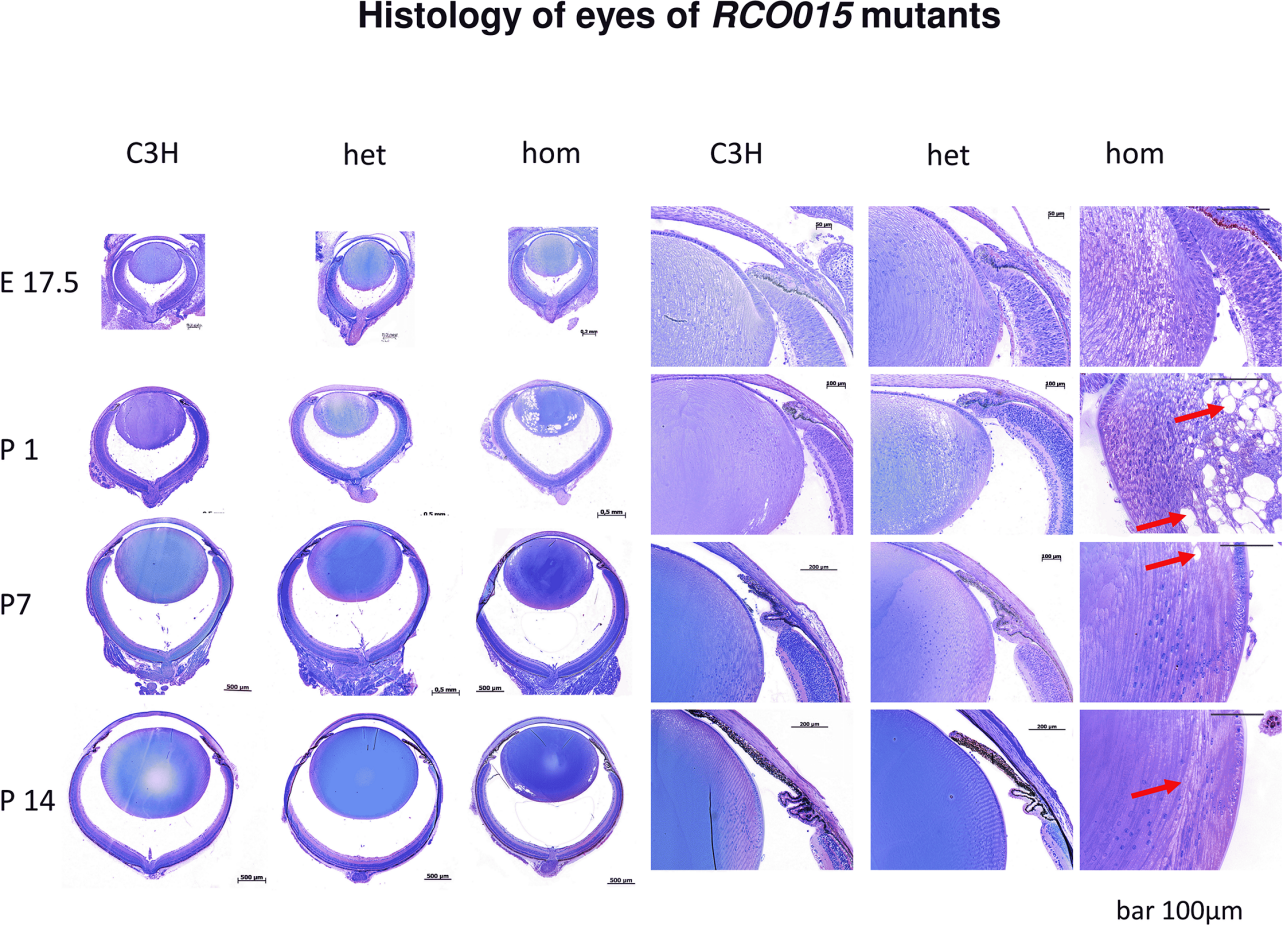
Histology. Histological sections from embryonic day 17.5 (E.17.5) till postnatal day 14 (P14) are given in an overview (left panel) and in a higher magnification of the anterior/equatorial region in the right panel of wild types, heterozygous and homozygous mutants. Cataract formation starts after birth in the homozygous mutants only. The first signs are vacuoles in the posterior cortical region of newborn mice (P1, red arrows), which are compressed later by the growing lens (P7, red arrow). Finally, they give rise to the sharp boundaries in the cortical region (P14, red arrow) being observed in the isolated lenses (Fig 2A). The bars indicate the magnification of the individual figures; the lenses of the homozygous mutants are shown in an even higher magnification than the heterozygotes to demonstrate the pathological processes in more detail.

By histological analysis of the developing mutant embryos (Fig 5) it is obvious that the lens is clear till the end of embryonic development (E17.5) in all three genotypes. After birth, first signs of cataract formation became visible in the homozygous mutants as vacuoles at the posterior-subcapsular region at P7 (red arrows). One week later, the vacuoles are smaller and at P14 they almost disappeared. Instead, the cortical-equatorial region of the homozygous mutants looks different from the same area in the heterozygotes and in the wild type: a clear border is obvious to the deeper fiber cells which are seemingly intact. This border does not demarcate the organelle-free zone, since cell nuclei are still present on the other side before they eventually disappear. This borderline might rather correspond to the cortical rings observed in the isolated lenses (Fig 4A) and is understood as a consequence of the previous vacuoles compressed by the prenatal growth of the lens.

**Fig 6:**
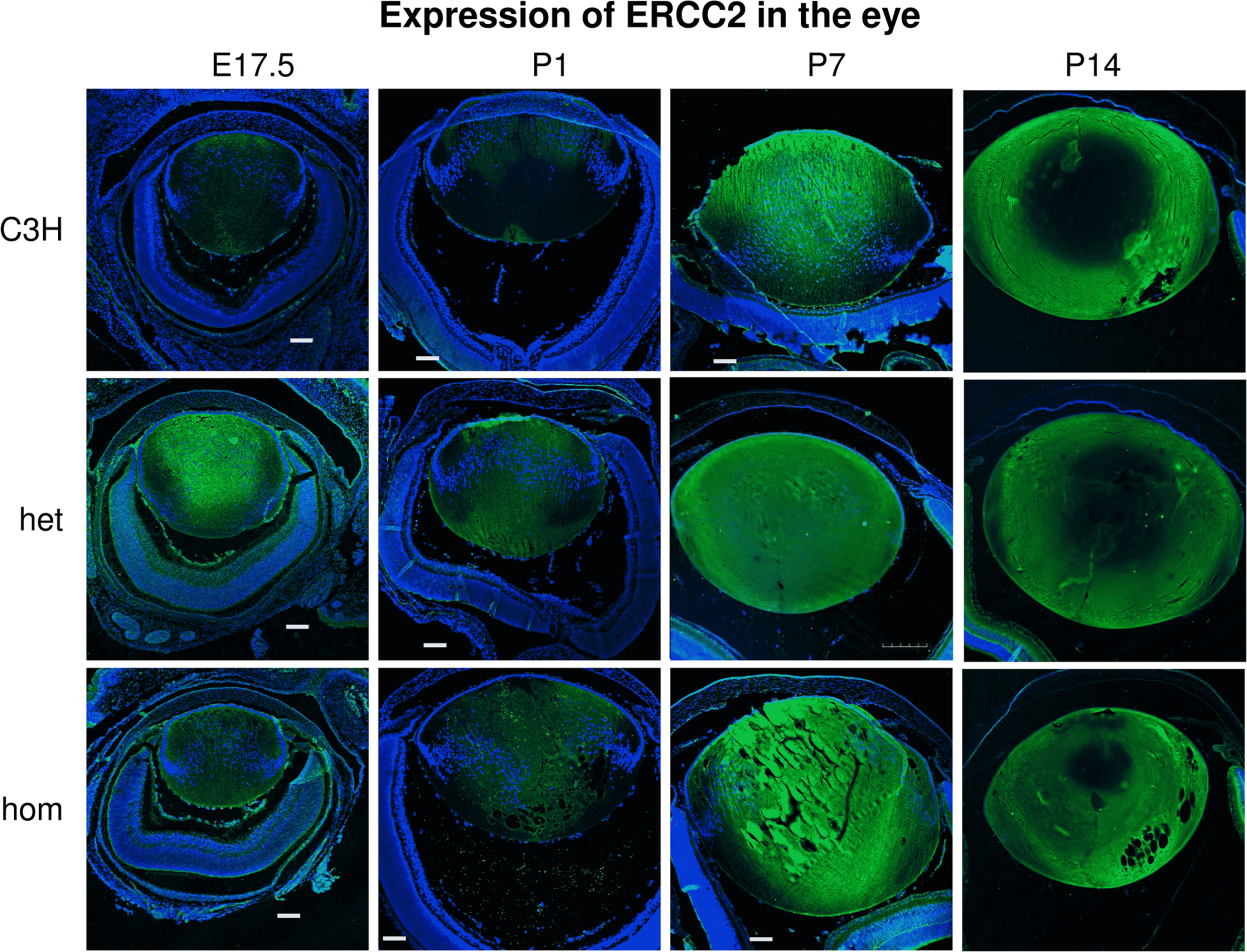
Immuno-histochemistry ERCC2 expression. Using antibodies against ERCC2 and Alexa488 (green) as secondary antibody, we demonstrate that ERCC2 is expressed weakly in the ocular lens around birth; expression is increasing from P7 onward. Cell nuclei are counterstained by DAPI (blue). ERCC2 is mainly expressed in the cytosol.

Immuno-histochemistry using antibodies against ERCC2 (Fig 6) demonstrated that ERCC2 is expressed in the ocular lens at E17.5 at the anterior and posterior parts of the primary lens fiber cells; it is not present in the cell nuclei, even if it is understood as a helicase and a DNA-repair enzyme [10]. This feature remains similar directly after birth (P1), but later (P7) it spreads out to the center and anterior part of the lens. At P14, the ERCC2-expression area is restricted to a cortical ring, with an ERCC2-free zone in the lens nucleus and the equatorial bow region. This expression pattern is roughly the same also in *RCO015* mutant mice, but the expression seems to be stronger in the mutants leading to a smaller ERCC2-free zone in the lens nucleus.

### Epigenetic marks in differentiating lens fiber cells

**Fig 7:**
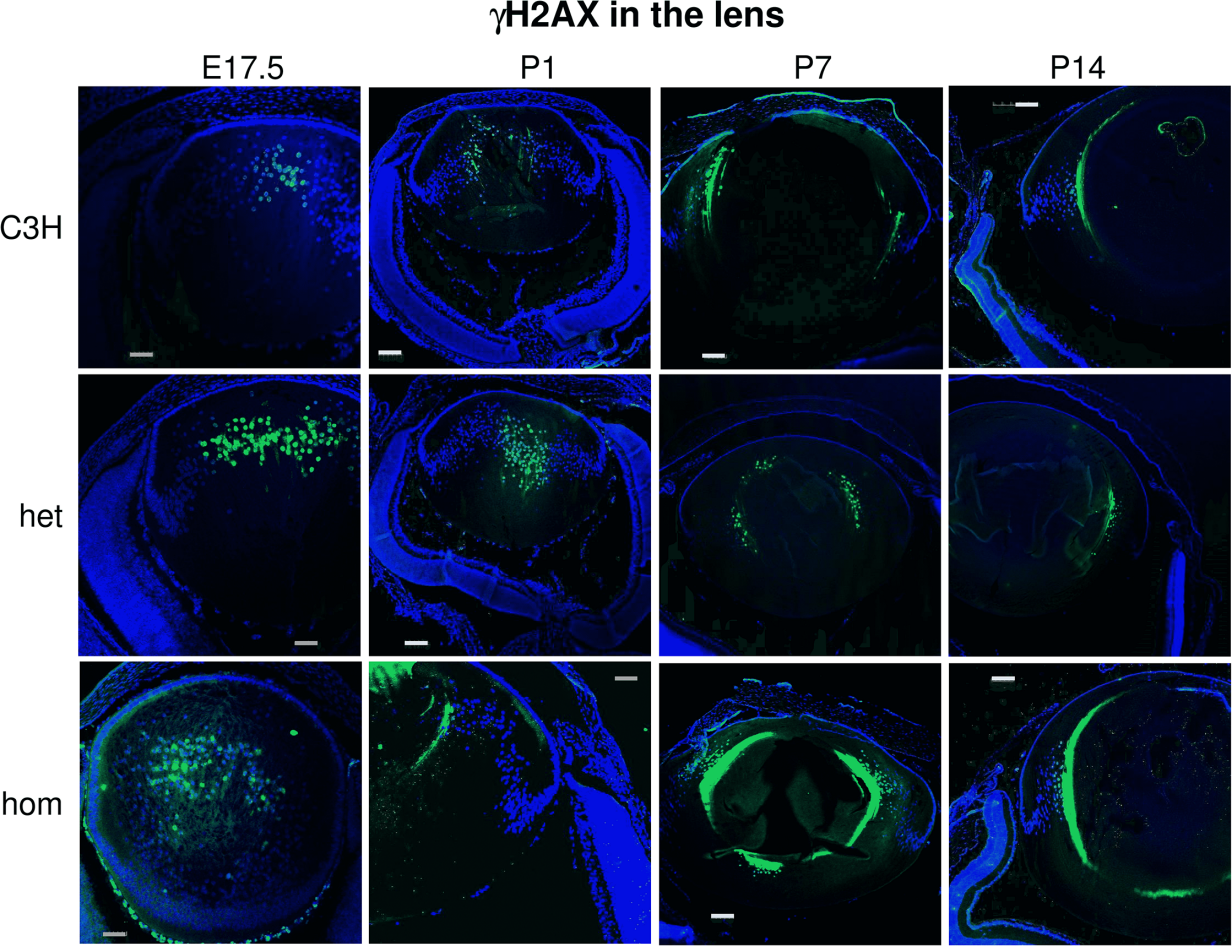
γH2AX: Immunohistochemistry. Using antibodies against γH2AX and Alexa488 (green) as secondary antibody, we demonstrate that γH2AX is expressed, where DNA degradation during terminal differentiation of lens fiber cells occurs; these sites represent DNA double strand breaks. Cell nuclei are counterstained by DAPI (blue); presence of γH2AX in the cell nuclei is indicated by overlapping colors of blue and green resulting in yellow.

The terminal differentiation of primary and secondary lens fiber cells needs degradation of the nuclei of these cells [20]. During this process, double strand breaks occur as indicated by the presence of γH2AX (the phosphorylated form of the histone H2AX [12]) in nuclei of primary lens fiber cells (Fig 7). At E17.5, the cell nuclei of the primary fibers are being degraded, and γH2AX is expressed in the anterior center of the lens. At P1, the expression pattern of γH2AX is shifted rather cortically, when the cell nuclei of the innermost secondary fiber cells are degraded. From P7 on, the expression pattern of γH2AX is restricted to a very sharp boundary at the transition to the organelle-free zone. In homozygous *RCO015* mutant mice, the expression pattern of γH2AX is roughly identical to the wild type, but broader, and the formation of the cortical ring appears earlier.

### Residual damage on irradiated DNA after repair

**Fig 8:**
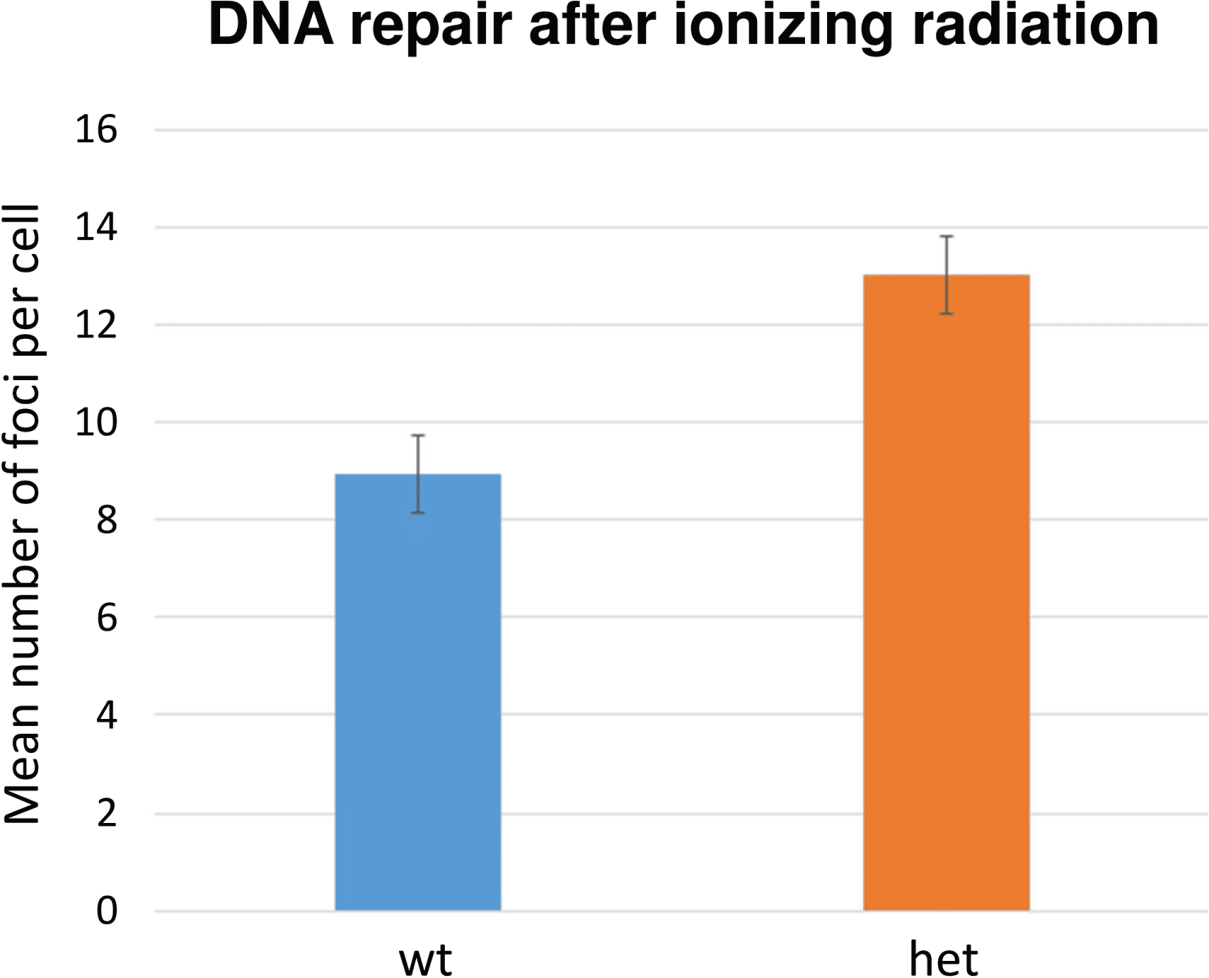
DNA repair after ionizing radiation. The remaining DNA damage after irradiation of isolated peripheral mouse lymphocytes with 1 Gy (^137^Cs) is given. 77–95 cells of each mouse were counted manually for remaining γH2AX foci 6 hrs after irradiation. It is obvious that the heterozygous mutant show significantly more foci than the wild types indicating that the repair of DNA double-strand breaks in the mutants is not as efficient as in the wild types (p = 0.006602). The columns represent the means of foci per nucleus from 3 males of each phenotype; bars indicate the standard error of the mean (SEM).

γH2AX is an early marker of DNA double strand breaks. Residual γH2AX foci are an indicator for remaining unrepaired DNA damage, thus for the individual DNA repair capacity. As demonstrated in Fig 8, there is a significant difference between residual damage of wild type DNA vs. heterozygous mutants after 1 Gy irradiation and 6 h repair time (p = 0.006602). The residual damage in peripheral lymphocytes of heterozygous mutants is about 1.5 times higher than in the wild type.

## Discussion

Here we report for the first time a mouse *Ercc2* mutant suffering from recessive cataracts. The founder mutant has been identified in a recessive ENU-screen because of their rough hair and smaller eyes; subsequent analysis identified the cataracts. The *Ercc2* mutation was characterized using a classical positional cloning approach combined with exome sequencing; the mutation was confirmed by co-segregation in the breeding colony and by its absence in other wild-type strains; there is no SNP reported at this site (ENSEMBL release 78, 2014).

Previously, several other *Ercc2* mutant alleles have been demonstrated in the mouse. Besides two different knock-out lines, also four mutant lines with targeted point mutations have been reported: G602D (twice, one of them is hypomorph); R683W and R722W [8, 21]. Our *RCO015* mutation affects the protein at its most C-terminal part (S737P). Most of these mutants show reduced body weight, increased mortality and ageing as well as increased tumorigenesis; reduced female fertility was observed only in the R722W mutant (Mouse Genome Informatics database, www.informatics.jax.org). Among the already known mutations, only the compound *Xpd* ^*G602D*^ / *Xpd* ^*XPCS*^ mutation was reported to affect the eye; in this case, corneal opacities have been observed in homozygous *Xpd* ^*G602D*^ mice [22].

Moreover, even in epidemiological studies, the association of *XPD* mutations with the prevalence of cataracts is discussed controversially: Ünal et al. [23] discussed a SNP at aa pos. 751 (Lys/Gln; rs13181). The Gln-allele (minor allele frequency: 24%; ENSEMBL; release 77, Oct 2014) might prevent cataract (case-control study; gene-dose dependent); however, Luo et al. [24] and Zhang et al. [25] did not confirm a statistically significant difference concerning the same SNP and using the same study design. Moreover, Padma et al. [26] reported another SNP (Asp312Asn; rs1799793; minor allele frequency 19%) being associated with a significant risk for cataracts [OR 1.97 (1.06–3.63)]. Our finding that ERCC2 is expressed in the lens (Fig 6) and that the S737P mutation leads to a recessive form of cataracts makes it very likely that the association of *ERCC2* SNPs with cataracts holds true also in human populations.

Mutations in *Ercc2*/*ERCC2* are involved in UV-induced cancer, Xeroderma pigmentosum subgroup D. Therefore, the gene symbol *Xpd* (for the mouse) or *XPD* (in humans) is used frequently. In the human population, some SNPs in the *ERCC2* gene are also associated with cancer (rs13181, see above, and rs238406), particularly with an increased risk of esophageal squamous cell carcinoma (OMIM 133239; [27]). Mutations in *XPD*/*ERCC2* are also involved in other disorders besides Xeroderma pigmentosum, namely the Cockayne Syndrome (CS; OMIM 216400) and Trichothiodystrophy (TTD; OMIM 601675; for a review, see [28]). TTD mice were found to exhibit many symptoms of premature aging including infertility [29]. In aged female rats, a significant decline of mRNA levels of *Ercc2* and *H2afx* (encoding H2AX) was observed in primordial follicle cells accompanied with a decrease of the phosphorylated form, γH2AX [30]. This might be an interesting observation in the context of the female infertility observed in the homozygous female mutants reported here.

Using our new model of the *RCO015* (*Ercc2* ^*S737P*^) mutant mice, we focused on the eye phenotype to find out how *Ercc2* is involved in the formation of cataracts. The lenses of the homozygous mutants are smaller (Fig 4A), and the cataracts are composed of a nuclear and a cortical component. Because of this kind of phenotype and since the ERCC2 protein is characterized as a DNA helicase involved in DNA replication and DNA repair, we tested the hypothesis whether ERCC2 might be involved in the terminal differentiation process of the ocular lens. This process is characterized by the degradation of cell nuclei and mitochondria including their DNA and by keeping the entire cell alive and at its place in the tissue for lifetime. This unique process was, therefore, in the focus of researchers since decades [20, 31, 32].

Our hypothesis that ERCC2 might be involved in the terminal differentiation process of lens fiber cells is supported by the expression pattern of ERCC2 in the lens (Fig 6): in the wild-type lens, ERCC2 protein mainly expressed two weeks after birth and is present in the central epithelial cells as well as in the nuclear fiber cells. Interestingly, one week later the center of the lens is free of ERCC2, but it is now present in the cortical region of the lens with a sharp border to the organelle-free zone (OFZ). Correspondingly, we checked the expression of γH2AX as an indicator for the presence of DNA double-strand breaks in the lens [12; 33]. γH2AX foci can be observed in the central part of the lens around birth (at E17.5 and at P1) indicating at these stages the presence of DNA double strand breaks—however, it is unlikely that they will be repaired since the DNA is going to be completely digested by DNases (primarily by DNase IIb [34]). At later stages (at P7 and 14), γH2AX is present in a small area just at the outermost cortex of the lens. Compared to the expression of ERCC2, γH2AX is involved earlier in the terminal differentiation process, both for the primary fiber cells in the lens nucleus as well as for the secondary fiber cells in the cortical area. In the *Ercc2* mutants, the expression pattern of ERCC2 and γH2AX is similar like in the wild types, however in both cases the homozygous mutants express these proteins in a broader area.

In this context it might be interesting to note that ERCC2 is not only involved in NER, but also in transcription-coupled nucleotide excision repair (TCNER; for a review see [35]). Cells with high transcription rates exhibit substantial levels of DNA double-strand breaks [36], and γH2AX was also observed to be present at longer single-stranded gaps possibly generated by strand displacement during transcription [37]. Therefore, γH2AX formation in cortical lens fiber cells might indicate also a high transcription rate in these cells. However, γH2AX is expressed in the area just before the organelle-free zone, i.e. in the area, where the final step of nuclear breakdown occurs, the digestion of DNA. At these stages it is difficult to assume that increased transcription will take place (as it would be in the more cortical fiber cells—however, these areas are free of γH2AX). Therefore, it is considered that the presence of γH2AX indicates here the presence of double-strand breaks promoting nuclear breakdown.

It is also an ongoing debate, whether mutations in *XPD*/*ERCC2* might also lead to an increase in sensitivity against ionizing radiation. In a previous study using a proliferation-based assay, primary mouse dermal fibroblast (MDFs) prepared from the tail skin of adult mice were treated with ionizing radiation up to 6 Gy; no differences in sensitivity to ionizing radiation were observed in any of the mutant cells relative to the wild-type MDFs [8]. Our study on lymphocytes isolated from blood, however, clearly indicate that even the heterozygotes are more sensitive to ionizing radiation at a dose of 1 Gy; particularly, radiation-induced DNA double-strand breaks are not as efficiently repaired as in wild types. Since humans heterozygous for *XPD*/*ERCC2* mutations are seemingly healthy, it is an important finding which has to be considered during radiation therapy.

From the results demonstrated here, we conclude that the mutation in the *Ercc2* gene leads to a delay in terminally differentiation of the primary and secondary lens fiber cells as indicated by the broader expression pattern of γH2AX. In case of the primary fiber cells, it is involved in the formation of the nuclear cataract, and with respect of the secondary fiber cells, it contributes to the cortical cataract. The formation of the cortical vacuoles in the lens is a transient state during the first two weeks of life. γH2AX is also more frequently observed in irradiated peripheral lymphocytes of heterozygous mutants indicating a higher radiation sensitivity of these mutants.
